# An Intricate Review on Nutritional and Analytical Profiling of Coconut, Flaxseed, Olive, and Sunflower Oil Blends

**DOI:** 10.3390/molecules26237187

**Published:** 2021-11-26

**Authors:** Roshina Rabail, Muhammad Asim Shabbir, Amna Sahar, Antoni Miecznikowski, Marek Kieliszek, Rana Muhammad Aadil

**Affiliations:** 1 National Institute of Food Science and Technology, University of Agriculture, Faisalabad 38000, Pakistan; roshina.rabail@hotmail.com (R.R.); asim-shabbir@live.com (M.A.S.); amnasahar@uaf.edu.pk (A.S.); 2 Department Food Engineering, University of Agriculture, Faisalabad 38000, Pakistan; 3 Department of Fermentation Technology, Waclaw Dabrowski Institute of Agricultural and Food Biotechnology, State Research Institute, 02-532 Warsaw, Poland; antoni.miecznikowski@ibprs.pl; 4 Department of Food Biotechnology and Microbiology, Institute of Food Sciences, Warsaw University of Life Sciences—SGGW, Nowoursynowska 159 C, 02-776 Warsaw, Poland

**Keywords:** blending vegetable oil, coconut oil blends, flaxseed oil blends, olive oil blends, sunflower oil blends

## Abstract

Vegetable oils (VOs), being our major dietary fat source, play a vital role in nourishment. Different VOs have highly contrasting fatty acid (FA) profiles and hence possess varying levels of health protectiveness. Consumption of a single VO cannot meet the recommended allowances of various FA either from saturated FA (SFA), monounsaturated FA (MUFA), polyunsaturated FA (PUFA), Ω-3 PUFAs, and medium-chain triglycerides (MCTs). Coconut oil (CO), flaxseed oil (FO), olive oil (OO), and sunflower oil (SFO) are among the top listed contrast VOs that are highly appreciated based on their rich contents of SFAs, Ω-3 PUFAs, MUFAs, and Ω-6 PUFA, respectively. Besides being protective against various disease biomarkers, these contrasting VOs are still inappropriate when consumed alone in 100% of daily fat recommendations. This review compiles the available data on blending of such contrasting VOs into single tailored blended oil (BO) with suitable FA composition to meet the recommended levels of SFA, MUFA, PUFA, MCTs, and Ω-3 to Ω-6 PUFA ratios which could ultimately serve as a cost-effective dietary intervention towards the health protectiveness and improvement of the whole population in general. The blending of any two or more VOs from CO, FO, OO, and SFO in the form of binary, ternary, or another type of blending was found to be very conclusive towards balancing FA composition; enhancing physiochemical and stability properties; and promising the therapeutic protectiveness of the resultant BOs.

## 1. Introduction

While planning to live protectively in the double burden of communicable diseases (CDs) and non-communicable diseases (NCDs), we have to change some dietary patterns. Among those, the selection of appropriate fat sources is the most important. Dietary fats, either from animal or plant origin, are the main determinant behind the epidemiology and negative pathophysiology of both CDs and NCDs [1]. It is the type of dietary fat that is responsible for the modulation of immune cell functioning, as it can alter the fatty acid (FA) content of immune cells, which appears to mediate the inflammatory process and predict future morbidities. The use of polyunsaturated fatty acids (PUFAs) is important in mediating inflammation [2]. Therefore, infectious and inflammatory diseases can be mediated by modulating the dietary FAs that influence the population of Gram-negative bacteria and changing the epithelial linings. Therefore, we should prepare to live accordingly, especially during the COVID-19 pandemic, by altering our diets to prevent the most severe inflammatory symptoms from developing. Lowering the ratio of saturated fatty acids (SFA) to monounsaturated fatty acids (MUFA) along with lowering total fats is a typical dietary intervention method to reduce chronic inflammatory illnesses [1].

Among NCDs, cardiovascular diseases (CVDs) and cancer are the main causes of global mortality, with lifestyle and dietary habits being the most important contributors [3]. Modern society’s high intake of inappropriate cooking oils is thought to be the leading cause of CVDs, as the impact of vegetable oil (VO) is determined by its FA content [4]. Imbalanced FA consumption is not only a cause behind CVDs fatality but is also linked with many other metabolic diseases such as nonalcoholic fatty liver disease (NAFLD), obesity, hypertension, etc. Both CVD and NAFLD share similar pathophysiological processes such as altered lipid metabolism and systemic inflammation, increased oxidative stress, and visceral adiposity which might explain their connection [5]. NAFLD progresses when the adaptive mechanism designed to prevent free FA (FFA) induced lipo-toxicity in the liver fails [6]. Consumption of lipids derived from plants may be advised to reduce mortality from CVDs [7]. Another study reported that the FA content of various dietary sources impacts the incidence rate of CVD. A diet high in MUFA: oleic acid (OA), as well as PUFAs: eicosapentaenoic acid (EPA), and docosahexaenoic acid (DHA) decreased the risk of CVDs. Therefore, dietary interventions are the major CVD prevention methods [8]. Hepatic disorders are also among the major causes of global morbidity and mortality. The rising prevalence in Asian nations is motivating researchers to look for natural preventative measures. To prevent chemical hepatic damage, it is more practical to stick to a healthy routine of eating VOs [9].

Therefore, high risk of mortality and disease progressions reinforce the need for clinicians to identify patients at increased risk and highlight the urgent need for the implementation of healthy dietary and lifestyle interventions [10]. Recommended dietary modifications for health include a lesser intake of SFA, trans-fats, simple carbohydrates, and animal proteins and more MUFAs, PUFAs, omega-3 PUFA (Ω-3 PUFA), plant-based proteins, and dietary fibers [11]. Such dietary interventions can only be achieved by consuming VOs of healthy and balanced FA profiles [12]. SFA, unsaturated FA (UFA), phosphatides, pigments, sterols, and tocopherols are all found in VOs. But for human nutrition, the ratio of SFA to UFA is critical [13]. Therefore, blending of VOs based on their FA profile diversity can help to design remarkably improved FA combinations and can play a key role in reducing the global burden of CD and NCDs. So far, this study is therefore compiled to combine all recent efforts carried out to develop VO blend with more profound health and economic benefits. This review will assist the oil industry to develop or design single oil formulations with improved FA profiles and suitability for human consumption using existing edible oils of highly distinct characteristics.

## 2. Vegetable Oils (VOs)

Oil and food are two important requirements of global economic development and people’s everyday lives. Excessive variations in either can drastically endanger global energy and food security [14]. FAO estimates that the world food markets are considered adequately balanced as long as enough wheat, corn, and oilseed outputs are available. The value of oilseeds is hard to overestimate in the national economy, due to their progressively growing food and non-food utilization in the world. Vegetable oils (VOs) made from oilseeds are essential for human nutrition and are widely employed in a variety of industries [15]. The output of VO has grown from 177.18 million metric tons to 198.68 million metric tons from 2014/2015 to 2017/2018 [16]. Global VO production is predicted to rise by 4% in all nine main oils (palm, soybean, rapeseed, sunflower, palm kernel, peanut, cottonseed, coconut, and olive) with respective advances in their consumption trend [17]. The nutritional value and quality characteristics of VOs are the most essential variables to consider in the food business. There is no single pure edible oil that has the oxidative stability (OS) and nutritional characteristics required, both of which cannot be achieved simultaneously by fortification alone [18]. A proper essential FA (EFA) ratio plays a vital part in sustaining optimum health. There is no such thing as a pure oil with an optimum FA composition and OS [19]. The nutritional quality and OS of edible oils are the two most important aspects to consider while evaluating them. Pure oils do not have an optimal FA composition or enough OS during processing or storage [20] and hence require special interventions to fill out nutritional and physiochemical deficiencies.

## 3. Need for Blending of Dietary Fats/Oils

The proportions of SFA, MUFA and PUFA in dietary fats should be 1:1.5:1 according to the World Health Organization (WHO) [21]. Cooking oils are one of the major nutritional sources consumed daily in today’s society. Oils are available in the market in combinations of two or more oils to provide the most health advantages. The pathogenic or protective effects of these oils are determined by a variety of parameters, including the content of the FAs, the duration and extent of heat exposure, daily intake, and consumption duration [3]. Fats and oils are important nutrients in the diets of both humans and animals since they are the dense source of energy and fat-soluble vitamins. The compositions of several frequently used Vos, as indicated by previous research, revealed that there is no single VO that has the appropriate amounts of FA needed to keep the cardiovascular system healthy, show an imbalance in their FA content, and that is not under the expert committee recommendations advised by nutritionists [4,21]. Since the health and nutritional elements of edible oils in meals and food products are gaining attention, it is becoming increasingly necessary to develop novel VO compositions with enhanced stability and nutritional value [22]. A simple and alternate technique is to create a physical combination of multiple oils in the appropriate proportions or to create structural lipids from diverse oils [21]. The blending of oils is a new method that may improve the antioxidant potential of the oil [23]. Nutritionists say that the correct blend of FAs in triacylglycerol (TAG) of oils and fats cannot always be reached by single oil. As a result, combining different types of oils is the greatest option for producing healthy VOs that can aid in the prevention and control of illnesses caused by FA imbalances. Blended oils (BO) consumption rich in OA and Ω-3 PUFA may help to correct the FA imbalances found in modern civilization diets, which are high in Ω-6/Ω-3 FA ratios and SFAs [4].

Blending edible oils has been developed as a cost-effective method of enhancing the organoleptic and physicochemical properties of VOs, as well as their oxidative stability [24]. Blending VOs is one possible option for optimal FA content and storage stability. Blending is an excellent option for producing edible oils with desirable properties and ensuring their quality. Oil blending decreases the peroxide value, FFA value, and acid value of the oil [25]. The BOs exhibit a wide range of applications due to their high Ω-9 contents as compared to single oils. They promote brain growth and lower the risk of heart disease [26]. The functional BO with a high OA and a low Ω-6/Ω-3 PUFA ratio enhanced growth metrics as well as lipid metabolism, oxidative stress, inflammation, blood pressure, blood lipids, and cardiovascular function. Their findings contributed to the development of novel dietary strategies for the prevention and treatment of high blood pressure and cardiovascular risk [4]. The blending of edible VOs is therefore one of the most cost-effective and straightforward methods for improving the nutritional, physical, and sensory qualities of any oil. A better blend with the overall higher quality might be produced based on the compositional diversity in oils [21]. The blending of different oils may pose a slight increase to the final product’s cost but cannot compete with the nutritional and clinical cost-effective outcome as highlighted in this review.

Pure oils do not have an optimal FA composition or enough OS during processing or storage [20] and hence need special interventions to fill out nutritional and physiochemical deficiencies.

## 4. Most Suitable Oils for Blending

**Figure 1:**
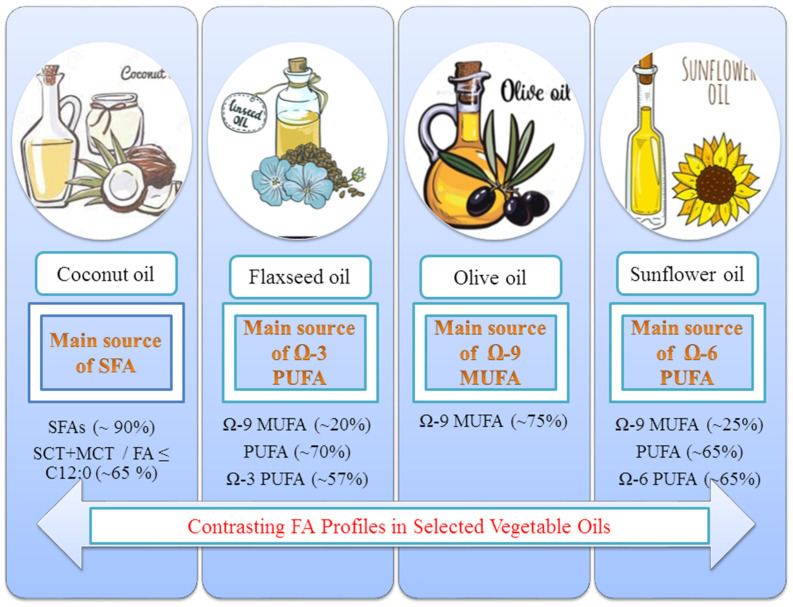
Selected vegetable oils with contrasting fatty acid profiles.

Based on global yields, oils of palm, sunflower, and soybean are the primary drivers, with a major increase in consumption trends by 4% towards sunflower and olive oil. Despite price increases of more than double in the last year, the use of sunflower oil (SFO) is expected to be at an all-time high, as prices of alternative VOs rise at a comparable rate. In terms of olive oil (OO), exports and consumption are expected to reach new highs due to increased customer desire for its use in home cooking. Furthermore, unlike other cooking oils, OO costs have not surged in the last year, encouraging increased food usage consumption [17]. Besides being majorly important in consumer demands, these two oils are also important for their contrasting FA profiles. This unique feature of being different or contrasting in FA composition has been used in many such previous interventions of blended oils (BO) as discussed later in this review. Therefore, SFO and OO were selected primarily to be overviewed here under the umbrella of oil blending, while coconut oil (CO) and flaxseed oil (FO) were also considered based upon their distinctive FA profiles. These four oils from vegetable origin have been selected based on their special attention-seeking characteristics, contrasting FA profiles (Figure 1), popularity, wide range of health protectiveness, and application. For example, CO was selected as a source of SFAs of high quality and health-protective in nature (i.e., medium-chain triglycerides/fatty acids (MCTs/MCFAs)), which have anti-obesity characteristics due to their easy and fast digestion in the body. The OO, on the other hand, contains a large level of MUFA and hence has superior frying qualities and stability. The FO, being a rich source of alpha-linolenic acid (ALA) Ω-3-PUFA is widely recognized for its anti-obesity, anti-diabetic, anti-carcinogenic, and cardio-protective qualities. On the other hand, SFO possesses the highest content of PUFA (68–72%) and has numerous positive health potentials [21]. There was no recent study available with compiled results from researches on the blending of these selected four edible oils with the most distinct FA profiles and characteristics. The effects of addition, or supplementation, or blending of any of these oils and their outcomes on FA profile, physiochemical properties, and disease biomarkers are highlighted in this review.

## 5. Selection and Collection of Data

This review is designed to explore available scientific data on blending or mixing, or combining of any single or more of the oils from selected oils; and to interpret their study outcomes for their improvements in FA ratios, their therapeutic potentials, better sensorial and physicochemical characteristics, and shelf life, etc. Therefore, recent accessible experiments on the production of single BO from selected contrasting VOs are collected and studied in line with this scenario. For the collection of data, advanced search options on Google scholar, Science Direct, and Scopus has been performed from 2017 to September 2021 with keywords selected as “vegetable oil blend *” OR “vegetable oil mix *” AND “coconut oil” AND/OR “Flaxseed oil” AND/OR “Linseed oil” AND/OR “Olive oil” AND/OR “Sunflower oil”.

## 6. Nutritional Composition of Coconut, Flaxseed, Olive, and Sunflower Oils

### 6.1. Coconut Oil (CO)

Coconut (*Cocos nucifera* L.) oil, especially virgin coconut oil (VCO) has experienced rapid development in the food industry in recent years and is one of the most clinically researched edible oils [27]. CO is high in MCTs, and it has a long list of health claims. SFAs are abundant in CO, especially lauric acid (48%) and myristic acid (19%), as shown in Table 1. Lauric acid (C12) is a major MCT in CO that is quickly absorbed by the liver and oxidized for energy. CO has been compared to other MCTs with special properties that distinguish it from other SFA-rich VOs such as palm oil due to its high amounts of lauric acid. CO can greatly increase high-density lipoprotein cholesterol (HDL-C) as a comparison to vegetable and animal oils. When compared to other VOs, CO increased low-density lipoprotein cholesterol (LDL-C) as well without affecting triglycerides. In this way, the virgin source of CO was found to have better lipid profiles [28]. CO’s growing success can also be attributed to its promotion of health benefits such as those related to CVDs, weight loss, Alzheimer’s disease, bone loss, diabetes, dental caries, and topical applications such as preventing atopic dermatitis and hair injury. Furthermore, CO use has struggled as a weight loss technique and cannot be used as a satiety-increasing substitute. If CO is to be used in the diet, it should be kept under the SFA guidelines of up to 10% of total calories. Still, long-term clinical trials are required to determine its significance [29].

An 8 week clinical trial of VOs supplementation on anthropometric and biochemical parameters in 75 obese women revealed that the CO group resulted in more weight loss and lowered BMI, percent body fat, waist circumference, and waist-to-height ratio in the anthropometric examination. Furthermore, the CO group demonstrated a greater decrease in glycemia and glycated hemoglobin biochemical markers [42]. In another study, CO consumption raised TC by 15.42 mg/dL, LDL-C by 10.14 mg/dL, and HDL-C by 2.61 mg/dL, and lowered glycosylated hemoglobin (HbA1c) by 0.39 mg/dL, but had no impact on triglycerides (TAG) (4.25 mg/dL) [43]. The MCTs of CO, as well as its high level of polyphenolic chemicals, which have been linked to its anti-inflammatory and improved metabolic properties, are regarded to provide the foundation for its potential health benefits [44]. Virgin CO and OO are among the top edible oils that contain antioxidants and help inhibit free radicals due to pancreatic beta-cell damage after alloxan injection and may lower the blood glucose levels while having no deleterious effects on the liver [45].

### 6.2. Flaxseed Oil (FO)

Flaxseed (*Linum usitatissimum* L.) or linseed oil (LO) has been thoroughly researched for its multiple health advantages, but it has yet to find a home in the cooking oil market. FSSAI and ICMR have recently recommended FO for cooking since it has a high quantity of fiber lignin, which works as an antioxidant. Blending FO is possible to minimize the high level of Ω-3 FA, which affects the oil’s stability [21]. Due to its high Ω-3 FA content as shown in Table 1, FO is utilized as a functional dietary component. Not only Ω-3 FA, but the presence of mono, di, and tri-terpenes such as β-Sesquiphellandrene, Copaene, Monoterpene, diethyl phthalate, Hexadecanoic acid, Squalene, and β-Sitosterol as well as ascorbic acid and gamma-tocopherol were found in the GC/MS analysis of FO [46]. FO is among the richest vegetative sources of alpha-linolenic acid (ALA), the main Ω-3 FA that has potent anti-inflammatory action, but is very oxidative and unstable to heat, so it can be used by blending with other oils [47,48]. Dietary FO reduced type II diabetes mellitus (T2DM) by reducing inflammation and altering the gut microbiota, which might help with diabetic dietary management [49]. Flaxseed, which is high in Ω-3 PUFA (ALA), lignans, proteins, and dietary fibers, has long been regarded as a valuable food component. FO, as one of the most useful components in flaxseed, may provide a variety of health advantages, including cardiovascular protection, anti-tumor, anti-inflammatory, life protection, diabetic therapy, and more [50].

### 6.3. Olive Oil (OO)

Olive (*Olea europaea* L.) oil is well known for its health advantages, and there is a rising trend in the intake of OO, particularly extra virgin olive oil (EVOO). These health benefits are primarily attributed to OO’s health-promoting nutrients (MUFAs) and non-nutrient such as polyphenols, tocopherols, and carotenoids, as well as its positive effects on the growth stimulation of the beneficial microorganisms, inhibition of foodborne pathogenic microorganisms, and antioxidant activity. These characteristics place OO among the top healthiest edible oils in the world [51]. EVOO is one of the most popular fat components in the Mediterranean diet, and it is recognized as a functional food due to its health benefits. It contains a significant amount of α-tocopherol, which is a component of the vitamin E complex [52]. Over 98% of OO is TAGs, with OA being the most abundant esterified FA (Table 1). OO is widely recognized for its health benefits, which are typically attributed to its phenolic components. The presence of nineteen phenolic compounds has been identified [40]. Due to the appreciable amounts of functionally active components such as phenolic compounds (e.g., tyrosol, oleocanthal, oleuropein, hydroxytyrosol, and oleuropein aglycone) and the presence of significantly bioactive carotenoids (provitamin A) such as β-carotene, and lutein, this OO (especially EVOO) can help prevent CVDs. Furthermore, using EVOO orally can help prevent cancer and type II diabetes. Its consumption can thus be advised not only for its healthy FA profile but also for the beneficial good effects of its bioactive components on human health [51]. Virgin CO and OO are among the top edible oils that contain antioxidants and help inhibit free radicals due to pancreatic beta-cell damage after alloxan injection and may lower the blood glucose levels while having no deleterious effects on the liver [45].

### 6.4. Sunflower Oil (SFO)

Sunflower (*Helianthus annuus* L.) oil is among the world’s five most significant VO crops. The ratio of Ω-3 to Ω-6 FA is critical for cardiovascular, heart, and other health advantages [53,54]. SFO is extracted from the seeds by either physical or chemical (n-hexane) extraction. This oil has a wide range of culinary applications, including cooking, baking, and frying. SFO is high in PUFA, linoleic acid (LA) (C18:2) (48.3–74.0%), and MUFA OA (C18:1) (14.0–39.4%) as listed in Table 1. PUFAs are more susceptible to oxidation, SFO’s high LA concentration will unavoidably impact its OS [55]. SFO is highly appreciated for its high concentration of PUFAs, as well as the presence of comparatively large levels of vitamin E and phytosterols. Bioactive sesquiterpene lactones have been recently discovered in non-germinated sunflower seeds and their presence is suggested in SFO as well [54]. Sunflower has been recognized as a functional food or nutraceutical due to its favorable health impacts, however, its full potential has yet to be realized. Sunflower has been found to have healing properties for a variety of illnesses in pharmacological studies. Sunflower has a variety of health advantages, including blood pressure and diabetes management, skin protection, cholesterol reduction, and other functions [56].

## 7. Effect of Blending on Nutritional and Physiochemical Properties of Oil

Many of the previous literature supports the blending of fats and oils for enhancing their nutritional and physicochemical properties. Table 2 enlists recent studies on blends of selected VOs (CO, FO, OO, and SFO) and their outcomes in terms of nutritional and physiochemical changes.

### 7.1. Binary Blends

**Figure 2:**
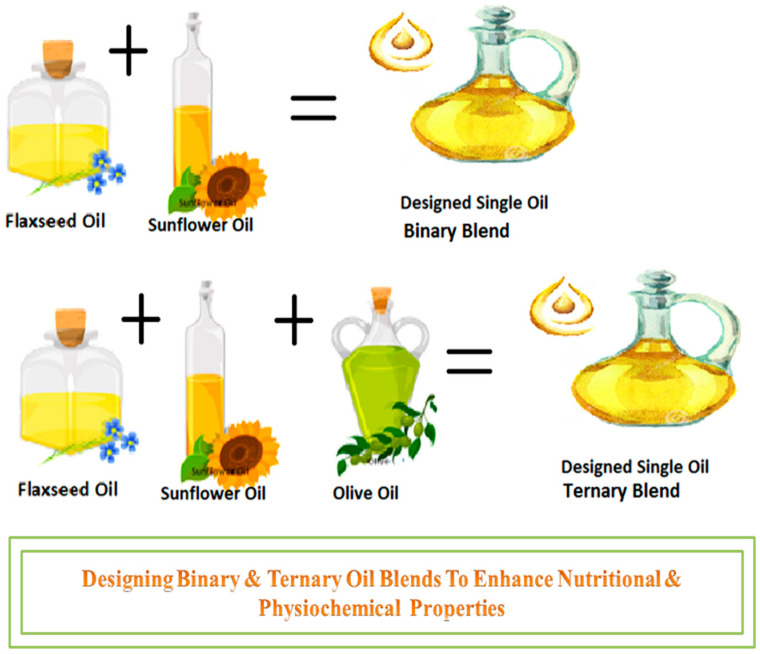
Binary and ternary oil blends.

Binary blends (i.e., blending of two different oils) had been a widely adopted strategy, primarily carried out to improve the deficiency of the main oil in that blend (Figure 2). In the most recent study binary blends with various ratios of refined SFO with cold-pressed FO to enrich it with Ω-3, PUFA were developed. With optimization of their ratio, BOs were created to have a high amount of Ω-6 and Ω-3 FAs. Total tocopherol content in BOs varied from 352.5 to 519.1 mg/kg, with carotenoids content ranging from 1.30 to 6.49 mg/kg. The presence of larger quantities of FO resulted in a rise in the content of ALA and carotenoids, as well as a decrease in total tocopherols, MUFA, and Ω-6 PUFA content, adding to the nutritional balance, with an overall higher quality blend oil formulation [57]. Similarly, binary blends of palm olein-based diacylglycerol (POL-DAG) and VCO were developed at different ratios to investigate physicochemical properties and thermal profiles. The composition of FA, acylglycerol composition, functional group, solid fat content, thermal performance, and iodine value (IV) were all thoroughly assessed. With the increasing concentration of POL-DAG, the DAG content increased and solid fat content decreased, raised the melting point, higher PUFAs, and improved techno-functional characteristics were reported [58].

The impact of combining SFO with pomegranate seed oil (PSO) from blanched seeds (95 °C/3 min) on the oil blends’ OS and antioxidant capabilities was investigated in a study, where total phenolics, total carotenoids, tocopherols, and FA composition of SFO and PSO blends in proportions of 90:10, 85:15, and 80:20 (*w*/*w*) were studied to determine the optimum blending ratio. An accelerated storage test was performed at 60 ± 2 °C for 20 days using the optimum blending ratio (85:15). Peroxide value, total oxidation value, and p-anisidine value as well as the depletion of the oils’ 2.2-azino-bis (3-ethylbenzothiazoline-6-sulfonic acid) (ABTS) and 2.2-diphenyl-1-picrylhydrazyl (DPPH) and radical scavenging activity, was determined. The OS of BOs was higher than that of SFO alone. The BOs also had a lower rate of DPPH and ABTS radical scavenging capability depletion than SFO, however, this did not differ substantially among oil blends [23]. Another study on binary blends with a better Ω-6 to Ω-3 PUFA ratio, high oxidative and thermal stability by mixing FO and POL. FO being rich in anti-inflammatory Ω-3 PUFA and highly oxidative was selected, whereas POL was selected due to its good thermal and OS and a low Ω-6 PUFA concentration. Blends of FO and POL with varying percentages (*v*/*v*) were created. Individual oils (FO and POL) were blended in prescribed proportions for 15–20 min using a mechanical homogenizer. Peroxide value, acid value, smoke point, percent FFA, para-Anisidine, and TOTOX values were used to assess oxidative and thermal stability. The nutritional content of the food was validated by measuring the FA composition. The nine-month storage stability of the BO was evaluated in terms of peroxide and POL acid value, as well as FA composition. An oxidative and thermal stability analysis, as well as a nine-month storage stability study, indicated that POL provided stability to the oil blend [48].

As VCO could not be used directly in the manufacture of edible fats due to its low fluidity and stiffness, necessitating the use of different modification procedures, therefore its physical blending with PO and chemical interesterification was carried out to enhance the functional properties. Before and after interesterification, the changes in chemical composition, slip melting point (SMP), solid fat content (SFC), and rheological characteristics (e.g., strain sweep, frequency sweep, flow behavior, and temperature sweep) of BOs were studied. All of the blends were trans-free, and the melting properties of BOs were altered via interesterification. All fat blends had a shear-thinning behavior based on rheological characteristics [27]. Binary blends of canola (CAO), olive, sesame (SSO) oils (i.e., CAO–OO and CAO–SSO) were rheologically tested. OO and SSO were separately mixed with CAO in the following proportions (by volume): 50:50, 30:70, and 10:90. Blending was accomplished easily by swirling the liquid for 5–10 min with a magnetic bead. The rheology was investigated at shear rates ranging from 1–132 s^−1^ and temperatures ranging from 15–65 °C before and after heating for 5, 10, and 15 min. The viscosity of the BOs changed linearly with its percentage content. The power law, the Sisko, Cross, and Carreau equations for viscosity-shear connection, and the Arrhenius and Williams– Landel–Ferry (WLF) equations for viscosity-temperature relation were all assessed. Short-term heating showed no influence on the rheological characteristics of pure oils or their mixes. Both the blends and the pure oils were found to be shear thinning. Their viscosities likewise dropped considerably as the temperature rose [60].

Binary mixing of oils was performed, taking into account the frequent use of CO, PAO, peanut oil (PNO), and groundnut oil (GNO). The oil was blended in 50:50 ratios with all four oils. This research highlighted the physicochemical qualities of BOs, such as pH, color, nutritional evaluation, and changes in BO parameters. These BOs were also used to make a variety of gram flour snacks such as pakoda, potato fries, and karasev. The shelf life of the snacks was investigated, taking into account changes in their synthetic and physical properties. The alterations seen in the oil extracted from the fried products were remarkably similar to those observed in the control. A physical examination of the prepared fritters revealed that they were synthetically safe to consume for up to 2 weeks. Among the BOs, CO with PNO had the most spreadability, while GNO with PAO had the least. The CO and GNO blend had the greatest ratio of UFA to SFA (58.8 %), PUFA: LA (18:2; 24.3%), ALA (18:2; 5%), OA (18:1; 25%), lauric acid (1.8%), myristic acid (1.6%), capric acid (2.8%), palmitic acid (16:0; 14.5%), and stearic acid (18:0; 9.2%). The PAO and PNO blend had the lowest UFA/SFA ratio (51.4%), including OA (21%), lauric acid (1.9%), and ALA (1.9%). CO with GNO blend had the best degree of acceptance for gram flour products of all the BOs. The mixture of PAO and PNO has a high concentration of FFA. The flour products made with CO and GNO revealed an acceptable appearance (8%). The presence of CO in blends was found best, yielding favorable effects with minimal increases in peroxide, free FA (FFA), IV, and saponification values (SV). Their work recommends that these blends may provide high-quality oils to extend the shelf life of food items in the future [26].

Another binary blending approach to balance FA composition between CO and SFO was carried out. As CO contains more than 90% SFA, with about 47% of these SFA is lauric acid, an essential SFA that provides excellent oxidation stability as well as other benefits such as anti-inflammatory characteristics. SFO has significant qualities for daily body balance; nevertheless, the high PUFA content offers limited OS. Knowing the nutritional characteristics of both raw materials, they were combined in two distinct proportions to assess FA composition: 50 + 50 (B1) and 70 SFO + 30 CO (B2). The acidity levels for both pure oils and blends are within Anvisa and Codex Alimentarius guidelines, and phenolic compounds were proportionate to the amount of SFO added into the CO. The oil stability index (OSI) values measured by Rancimat were high, particularly for CO, and the increase in OS in the mixes was related to the amount of CO added to SFO. The major cause of the increased OS of CO can be ascribed to the large quantity of lauric acid, an essential SFA. The combination of CO and SFO increased the OS of blends as well as the number of phenolic compounds, which serve as natural antioxidants and boost antioxidant capacity, as shown by DPPH tests [35]. A most recent study with the goal to create single oil that had a balanced ratio of SFA, MUFA, and PUFA, as well as better characteristics and shelf life using selected locally available common oils of linseed, coconut, soybean, olive, mustard, and sunflower. All of the oils were freshly extracted by using the cold-press methods. Oil mixtures were mixed in conical flasks for roughly an hour using a magnetic stirrer at a constant temperature of 40 °C. LO/FO was mixed with SFO, OO, CO, mustard oil (MO), and soybean oil (SBO), in different ratios, such as 80:20, 70:30, 60:40, 50:50, 40:60, 30:70, and 20:80, (*v*/*v*), respectively. All of the oils’ chemical characteristics and FA composition (Acid value, peroxide value, and iodine value) were tested before and after mixing, and the storage quality was determined by storing at room temperature. FA ratios (SFA:MUFA:PUFA) of 1.5:1:3.1 for LO and CO (80:20), 1:1.4:4.6 for LO and SBO (20:80), and 1:1.9:3.4 for LO and OO (80:20) were found to be near to the recommended intake (1:1.5:1) and exhibited superior quality across all blends. Except for a few blends (LO:SFO, LO:SBO, and LO:MO) due to their greater PUFA contents, the storage quality of the majority of blends preserved their quality and was judged appropriate for consumption. The above-mentioned resultant FA ratios of BOs were highly recommended for maintaining excellent health since no natural oil has such a balanced composition. These blends were overall found nutritionally superior as each oil has its own character and composition, and blending has increased the nutritional quality of samples [21].

A similar approach for VO blending to increase their uses and nutritional quality was performed based on a simple, quick, cheap, and non-destructive method for characterizing various types of VO blends using color histograms. Four binary edible oil blend regression model datasets performed were: POL-rapeseed, POL-SFO, SBO-SFO, and SBO-rapeseed. Despite the high coefficient of determination of the support vector regression (SVR) and Levenberg-Marquardt artificial neural network (LMANN) regression models in all of the aforementioned data sets, Bayesian regularized artificial neural networks (BRANN) provided better results up to 97 percent for HSI color histograms in both the training and test sets. The principal component analysis (PCA) technique was used to minimize the number of independent variables for modeling. Finally, the image analysis findings were compared to those produced by processing FT-IR spectra of BOs. The results showed that image analysis of BOs yielded results equivalent to those obtained by processing FT-IR spectra for edible oil characterization and indicate that the suggested approach has the potential to characterize various binary mixes of edible oils [18]. To improve the quality characteristics of refined SFO, 9 BOs were developed using LO/FO, grapeseed oil (GSO), and CO. The results for physicochemical properties revealed superior features for blends including lower acidic value and higher autoxidation stability. The best blend combination for SFO was with CO. Lovage leaves (*Levisticum officinale*) extract was used as an additive to improve properties and the preservation of blends. The resultant blends demonstrated higher quality characteristics and can be recommended for human consumption [36].

SFO when blended with cold-pressed black cumin (*Nigella sativa*) oil (BCO) at percentages of 5%, 10%, and 20% (*w*/*w*). The OS of SFO and blends was investigated during storage under thermally accelerated oxidation conditions. The oxidation process was tracked by measuring peroxide value, conjugated dienes, and conjugated trienes. The amounts of volatile oxidation chemicals, thymoquinone, and tocopherols in oils and BOs were also measured during thermal oxidation. Blending had no substantial effect on the FA composition of blends containing mostly LA and OAs. At the end of storage, inverse connections between peroxide value and OS were discovered. Blends were more stable than SFO, most likely owing to variations in the amounts of thymoquinone and tocopherols present in BCO. Blending edible oils has developed as a cost-effective method of enhancing the organoleptic and physicochemical properties of VOs, as well as their OS [24]. Cold-pressed VOs available in the consumer market (sunflower, rapeseed, soybean, flaxseed, camelina, mustard, hempseed, sesame, amaranth, cedar, walnut, pumpkin, wheat germ, EVOO, and grapeseed) were examined to design and validate blends with appropriate Ω-3:Ω-6 PUFA ratios. The blends’ autocatalytic oxidation was investigated at a storage temperature of 202 °C with free access to light and air. Blending 45% walnut oil with SFO resulted in lowered peroxides and FFA, and Ω-3:Ω-6 PUFA ratio similar to that suggested for daily consumption. The authors proposed therapeutic nutrition blends of VOs with a greater ratio of Ω-3:Ω-6 PUFA (75% SFO plus 25% FO). Blending conventional SFO with other types of VOs solves two problems: increasing the biological value of fat by optimizing the FA content and increasing resistance to oxidative deterioration. Such blends can be used to make health-improving products [32].

### 7.2. Ternary Blends

Blending of oils the in literature was not confined to binary blends only, but the blending of three, four, or even more edible oils or fats was also reported in some studies. In such blends, the purpose is not only confined to improving the nutritional quality or to meet deficiency but improvements in physiochemical, rheological, and storage properties can also be achieved. Most of the VOs do not meet the WHO’s recommended FA profile, but are rich in PUFAs, that are susceptible to lipid oxidation, which causes rancidity and reduces the oil’s nutritional value. Therefore, VOs can be customized to provide an antioxidant-rich BO with improved OS during accelerated storage. The OS of six blends from three selected oils including rice bran oil (RBO) 50–75%, PNO 20–40%, and FO 5–10% at various concentrations was investigated. RBO enhanced the amount of tocopherol and oryzanol in the blend. The OS of RBO-PO-FO: 75-20-5 was superior among all other blends [59]. As the diet with an appropriate PUFA ratio of Ω-6 to Ω-3 inhibits the development of many inflammatory disorders. Ternary oil blends with appropriate Ω-6 to Ω-3 ratios were created with OO, SFO, and cress (CRO) oils. The blends’ OS, thermal profile, FA composition, tocopherol contents, and physicochemical characteristics were investigated. Blends were made with ratios of 2, 3, 4, and 5, Ω-6 to Ω-3 ratio. The most important parameters influencing the oxidation and thermal stabilities of the oils were FA composition and tocopherol concentration. All of the blends had high-quality indexes. As a result, oil blends with high OS, antioxidant content, optimum Ω-6 to Ω-3 ratios, and suggested FA compositions may be created. Blending different qualities of OO, SFO, and CRO leads to new oils with better functional attributes and uses in final goods. All of the BOs had strong antioxidant levels, excellent quality indices, and good oxidative and thermal stability. The blends have a FA (SFA 10% FE, PUFA = 6% – 11% FE, and MUFA = 14% – 19% FE). Adult women and men can meet their daily fat requirements by consuming 77 g and 98 g of any of these blends, respectively, daily [33].

The influence of different types of deep-frying oils on the production of acrylamide in deep-fried potatoes was investigated. Straight sliced potatoes were deep-fried in palm oil (PAO), CO, RBO, VO blend B1 and B2 for 12 min at 150 °C and 170 °C as cycle 1 (12 min) and Cycle 2 (continuously deep-fried in the same oil). The acrylamide analysis technique includes solvent extraction and HPLC combined with DAD. In cycle 1, RBO had the lowest acrylamide level when compared to the control (PAO). The CO, on the other hand, demonstrated the most potential for usage for repeated deep-frying oil. According to the findings, mixing VO can give decreased acrylamide levels in deep-fried foods [62]. The chemical, nutritional, and rheological characteristics of BOs in three ratios of OO, SSO, and LO/FO, 65:30:5, 60:30:10, and 55:30:15, were investigated. The acidity, peroxide, rancimat test, FA profile, nutritional indices, and rheological characteristics were all determined. Atherogenic and thrombogenic indexes, as well as hypocholesterolemic: hypercholesterolemic, PUFA: SFA, and Ω-6:Ω-3 ratios, were assessed. The results showed that combining other VOs with LO may achieve a Ω-6:Ω-3 ratio. The findings revealed that prepared oils had an excellent combination of oxidation stability (OS) and nutritional characteristics. Rheological measurements revealed that these oil mixes behaved Newtonian at 4 °C and 25 °C. Conclusion: The addition of LO to VOs having high amounts of bioactive components was shown to be a simple and cost-effective method of producing a functional oil with good nutritional and stability qualities [20].

A mixed oil formulation was created to provide a product high in EFAs and with a lower ratio of linoleic and linolenic (Ω-6:Ω-3) FA. The formulas were optimized using multivariate mixture design methods. Three formulations of varying compositions were chosen and tested for OS, sensory acceptability, FA content, phytosterols, and tocopherols. The formulation with superior sensory acceptability was made up of 85% EVOO, 3% LO/FO), and 12% safflower oil, and it had the lowest ratio of linoleic and linolenic acids (Ω-6:Ω-3) as compared to the EVOO, higher OS, and higher amounts of phytosterols and tocopherols. The mixed oil formulation (blend) produced was nutritionally enriched and could be used as an ingredient in a variety of food items at household and industrial scale to enrich food items with high levels of EFAs, phytosterols, and tocopherols and a better balanced (Ω-6:Ω-3) FA ratio encouraging health advantages via consumption [64]. Physical, chemical, and nutritional characteristics of BO produced by combining FO with SSO and OO in three distinct ratios (60:30:10, 65:30:5, and 55:30:15). The FA composition, phenolic compound, peroxide, anisidine levels, and Schaal tests were used to assess the quality and physicochemical characteristics of these blends, which were kept at 4 °C and 24 °C. FA composition revealed that including 10% and 15% FO into blends resulted in an appropriate EFA ratio. The sample with 5% FO had the greatest phenolic concentration across treatments, and these chemicals decreased significantly after storage. The peroxide levels of all samples increased significantly after storage. Increasing the FO concentration of the blends increases the anisidine value. Blending SO and OO with FO resulted in oil blends with a favorable EFA balance. Although the peroxide and anisidine values of the oil blends rose during storage, the blends were of excellent quality for household and industrial usage [19].

### 7.3. Other Blends

A computer modeling of VO blends was performed using the brute force method. Biomedical requirements were developed, considering the required chemical composition, structural correlations of the biological value of blends according to the FA compliance (Ω-6 to Ω-3), and mass fractions of the main components of the product. An automated research system was designed and deployed to simulate the composition of mixes based on a specific goal function of the Ω-6 to Ω-3 PUFA ratio. Three options were acquired for a blend composition with Ω-6:Ω-3 in the first and second variants (5:1), and in the third-10:1, allowing them to be used for both healthy and therapeutic purposes [61]. Under accelerated storage at 60 °C/20 days, the OS of LO/FO, cotton (CTO), and CO oils, as well as BO1: FO-CO, BO2: FO-CTO, and BO3: FO-CTO-CO compound oils, was assessed. CO was found to be rather stable, owing to low amounts of peroxides, conjugated dienes, ρ-anisidine, and a lengthy induction time. Furthermore, it enhanced the stability of FO in the production of BO3 compound oil in conjunction with CTO. In terms of FA composition, the compound oils were found to be mostly constituted of UFAs. CTO and CO retained more total phytosterols after 20 days of storage, with 78.87 and 76.16% retention, respectively, as compared to LO. BO2 had the highest retention of total tocopherols at the end of storage (90.81%). In terms of antioxidant activity, the DPPH technique revealed that as storage time increased, antioxidant compounds of FO in, BO1, and BO3 oils decreased. Oscillations were found using the FRAP technique, particularly in linseed and compound oils. Even though the oils deteriorated with time, it was able to confirm that CTO and CO helped to enhance the stability of FO [63].

## 8. Effect of Blending on Therapeutic Potentials of Oil

**Figure 3:**
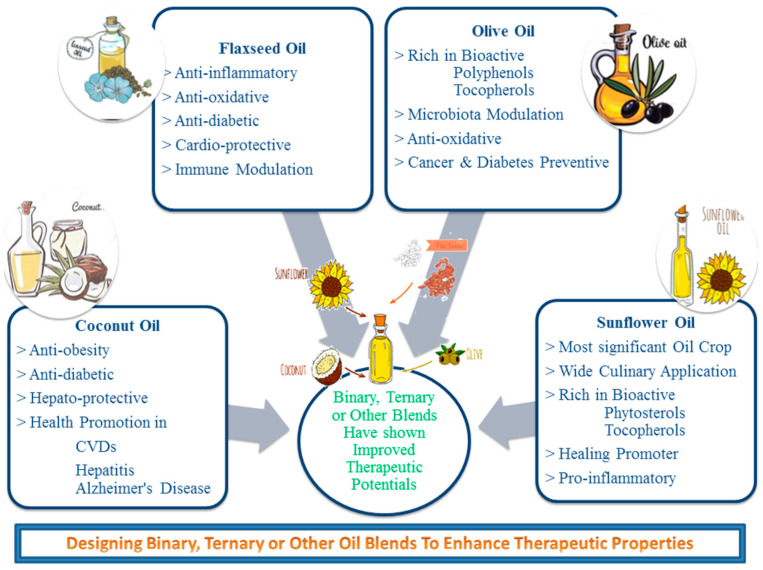
Effect of binary or ternary (CO, FO, OO, SFO) blending on therapeutical potential.

Recent studies on remodeling the therapeutical potential of BOs made from selected VOs (CO, FO, OO, and SFO) have been listed in Table 3 and their conclusive outcomes have been summarized in Figure 3.

### 8.1. Anti-Inflammatory Potential

The therapeutic potential of a binary blend of FO with PO with a better Ω-6 to Ω-3 FA ratio, high oxidative and thermal stability was investigated. The biological effects on cell survival, FA uptake, and inflammatory markers were investigated in the THP-1 cell line. According to the statistics, blending improved the Ω-6 to Ω-3 FA ratio. The FA profiles of cells treated with these blends revealed ALA uptake and a reduction in inflammatory TNF levels without compromising cell viability. Thus, FO and PO blend results in improved health benefits, nutritional profile, and stability [48]. The effects of a functional BO with high levels of OA (50.93%) and ALA (5.41%) on CVD health, body weight (BW), and blood pressure (BP) as compared to lard oil and PNO were evaluated. BO was made from oils of corn (CNO), canola, olive, sunflower, and PNO. Ninety male Wistar rats were split into three groups and given BO, lard oil, and PNO for twelve weeks. Each group was separated into three groups: low, middle, and high fat, with oil supply ratios of 6.7%, 10.9%, and 48.1%, respectively. BO treated rats, particularly those given a high dosage (HBO), had reduced mRNA expression of lipid anabolism-related genes, toll-like receptor 4 (TLR4), lipid inflammatory-related genes, nuclear factor kappa beta (NF-B), and monocyte chemoattractant protein (MCP)-1 while increasing mRNA expression of lipid catabolism-related genes and peroxisome proliferator-activated receptor-gamma mRNA (PPAR mRNA). Furthermore, BO supplementation reduced serum levels of CRP and TNF-α [4].

### 8.2. Anti-Obesity Potential

The effects of high-fat diets (HFD) with different ratios of CO and CNO with LA levels of very low 1% energy from LA, low 2.80% energy from LA, moderate 5.80% of energy from LA, and high 9.70% of energy from LA on fat deposition and selected metabolic biomarkers of male Sprague-Dawley rats were investigated. The initial and final BWs of the participants were recorded. After an eight-week feeding trial, adipose tissue, blood, and liver samples were taken for examination. The high-LA diet resulted in greater BW increase and epididymal fat deposition when compared to the very low-LA diet. There were no significant changes in the test diets’ liver-to-BW ratio, blood glucose, visfatin, and leptin levels. Serum TNF-α, insulin, and C-peptide levels do not increase appreciably as dietary LA levels rise. A high-fat meal including high amounts of LA caused BW gain and epididymal fat deposition in rats, but did not affect selected metabolic indicators, according to the findings. The blends with a low-LA, MCFA diet resulted in less body weight increase than the high-LA diet. The high-LA diet resulted in considerably more fat accumulation in the epididymis [65]. The effects of a functional BO with high levels of OA (50.93%) and ALA (5.41%) on BW as compared to lard oil and PNO were evaluated. BO was made from CNO, CAO, OO, SFO, and PNO. Ninety male Wistar rats were split into three groups and given BO, lard oil, and PNO for twelve weeks. Each group was separated into three groups: low, middle, and high fat, with oil supply ratios of 6.7%, 10.9%, and 48.1%, respectively. The BO treated rats, particularly those given a high dosage (HBO), had considerably reduced BW, fat weight, liver weight, fat ratio, food consumption, and energy intake [4].

### 8.3. Hepatoprotective Potential

Healthy adult male rabbits were split into four groups (*n* = 12), with two groups receiving 1 and 2 mL/kg/day of repeatedly heated mix VOs, the third receiving 1 ml/kg/day of single time heated mix VOs, and the fourth serving as controls. Serum liver function markers such as total protein, albumin, serum SGOT, SGPT, and ALP, as well as the activity of hepatic antioxidant enzymes such as superoxide dismutase (SOD), catalase (CAT), glutathione peroxidase (GPx), and malondialdehyde (MDA) for lipid peroxidation, were compared between rabbit groups. All four groups were subjected to histopathological examinations. In comparison to the control, high and low dosage mix VO treated groups had significantly higher hepatic enzymes and MDA levels but decreased total protein, serum albumin, GPx, SOD, and CAT levels. Accumulation of liver fat in low and high oil dose treated groups was verified by microscopic inspection of liver tissues, which revealed substantial fat accumulation in liver tissues, as well as 40–60% higher oxidative stress compared to control, in a dose-dependent way. These findings suggest that consuming thermally damaged blend VOs over an extended period can severely impair liver function and ruin its histological structure via fat formation and oxidative stress in both high and moderate dosages [9]. The hepatoprotective effects of OO, FO, and their blend on the hepatocytes of rats’ livers were investigated. The results showed that the diet containing OO, FO, and blend oils 100% substitution caused a significant decrease in TC, with values of 79.71,76.18, and 76.66 mg/dL respectively, when compared to the positive control group, while there were no significant differences in triglyceride levels between these three treatments. The livers of wounded rats revealed centro lobular coagulative necrosis, fatty alteration of the hepatocytes, and dilatation with congestion in the central vein following CCl4 treatments, according to histological analysis. The results showed that giving rats a baseline diet comprising 100% mixed oil resulted in the greatest liver improvement and dramatically reduced CCl4-induced necrosis and lymphocyte infiltration [68].

### 8.4. Cardio-Protective Potential

Compared to refined OO as a control, the effects of two oil blends produced from refined RBO, FO, and SSO, with various MUFA, PUFA, and Ω-3 to Ω-6 FA ratios and varied phytonutrient concentrations on blood lipid profile and other indicators of cardiometabolic health outcomes. A randomized controlled study was conducted on 143 borderline hypercholesterolemic volunteers aged 50 to 70 who had a BMI of ≤27.5. For all three intervention oils, the results showed significant reductions in TC, LDL, TAG, apoB, TC to HDL and apoB to apoA1 ratios, systolic BP, diastolic BP, and serum glucose levels, as well as a small significant increase in BW, implying that they can improve blood lipid profile and other cardiometabolic parameters [66]. The effects of a functional BO with high levels of OA (50.93%) and ALA (5.41%) on CVD health, blood pressure (BP), and BW as compared to lard oil and PNO were evaluated. BO was made from CNO, CAO, OO, SFO, and PNO. Ninety male Wistar rats were split into three groups and given BO, lard oil, and PNO for twelve weeks. Each group was separated into three groups: low, middle, and high fat, with oil supply ratios of 6.7%, 10.9%, and 48.1%, respectively. BO treated rats, particularly those given a high dosage (HBO), had considerably reduced body fat weight, liver weight, fat ratio, and energy intake. BO decreased Ω-6/Ω-3 ratios in plasma, liver, and adipose tissues, as well as serum triglycerides (TAGs) and LDL-C, but raised HDL-C. These data suggest that a novel mix of CAO, maize oil, OO, PNO, and SFO with a low Ω-6/Ω-3 PUFA ratio of 6:1 might help to prevent and manage CVDs [4]. In animals, serum lipid metabolic reactions are linked to specific metabolic diseases caused by dietary habits. Such connections, however, have not been seen in fish. Lipidomic studies were carried out to explore fish lipid metabolic responses to VO blends (VOB) and to identify the mechanisms through which dietary VOB influences serum lipid profiles. Dietary VOB has a significant impact on serum lipid profiles, particularly the triglyceride: phosphatidylcholine (PC) ratio, by blocking hepatic PC production and promoting hepatic and intestinal PC biosynthesis. In vitro studies showed that variations in serum TAG:PC ratios might be ascribed in part to dietary FA composition [70].

### 8.5. Anti-Oxidative and Immune Protective Potential

Effects of two new mixed plant oils (MPO) on weanling piglet performance, serum immunity, antioxidant capacity, and intestinal morphology to those of SBO were investigated. A total of 108 piglets, weaned at day 28 and weighing 8.80 ± 1.02 kg, were randomly assigned to one of three food regimens. The dietary treatments included a control diet (corn-soybean + 5% SBO in phase 1 or 4% SBO in phase 2), a MPO 1 diet (basal diet + 5% MPO1 in phase 1 or 4% MPO1 in phase 2) where MPO is a mixture of 10% CO, 15% CNO, 15% LO, 15% PNO, 20% PAO and 25% SO). The MPO diet exhibited higher average daily growth and feed efficiency in phase 1 and overall (d 0–28), as well as higher serum SOD concentration. On day 28, these pigs also exhibited greater serum IgG, SOD, glutathione peroxidase, villus height in the duodenum and jejunum, and apparent total tract digestibility (ATTD) of ether extract. According to the findings, MPO can be a superior energy source to only SBO in terms of enhancing growth performance, serum immunity, antioxidant capacity, apparent total tract digestibility of ether extract, and intestinal morphology in weanling pigs [67]. The effects of a functional BO (CNO, CAO, OO, SFO, and PNO) with high levels of OA (50.93%) and α-linolenic acid (ALA) (5.41%) in 90 male Wistar rats on a low, middle, and high-fat diet with oil supply ratios of 6.7%, 10.9%, and 48.1% enhanced the activity of antioxidant enzymes such as catalase (CAT), glutathione peroxidase (GPx), and SOD by decreasing malondialdehyde (MDA) and ROS in tissues [4]. Similarly, DPPH antioxidant activity, phenolic compounds, and OS of CO and SFO blends were analyzed and were found in the SFO used. The combination of CO and SFO increased the OS of blends as well as the number of phenolic compounds, which serve as natural antioxidants and boost antioxidant capacity, as shown by DPPH tests [35].

### 8.6. Growth and Other Potentials

*Nile tilapia* (fish) production in the subtropics is dependent on lower winter temperatures. Adequate FA nutrition of fish is a strategy to compensate for the lowering of the temperature. Three plant oil blends (CO, OO, SFO, and LO) and fish oil (FO) as a control were given to juvenile Nile tilapia for 9 or 12 weeks at optimum (28 °C) or suboptimal (22 °C) temperatures, respectively. The plant oil blends were designed to replicate the FA groups found in FO by changing the amounts of Ω-3 and Ω-6 PUFA. Tilapia given plant oil mixtures grew similarly at each rearing temperature [69]. DHA is a long chain Ω-3 PUFA found predominantly in marine fishes. Vegetarian diets do not contain preformed DHA, but they can obtain it from shorter chain ALA present in plant oils. In adults, the conversion efficiency of ALA to DHA is quite low. This may result in DHA deficiency in the vegetarian population. Curcumin, a diferuloylmethane found in the spice turmeric, has the potential to enhance the synthesis of DHA from ALA by activating the enzymes FADS2 and elongase 2. Curcumin was dissolved in CO, SFO, or LO, enriched in n-3 PUFAs), and nanoemulsions were created after mixing with LipoidTM in a high-pressure homogenizer. The nanoemulsions were offered to weaned rats with AIN-93 meals for 60 days. Curcumin levels in blood, liver, heart, and brain were shown to be high in rats fed a nanoemulsion containing curcumin in LSO. DHA levels in blood and tissue lipids increased significantly in rats fed LSO with curcumin nanoemulsions. As a result, supplementing diets with ALA-rich LO/FO and curcumin may enhance DHA concentrations in serum, heart, liver, and brain lipids, which may have consequences for satisfying the DHA needs of vegetarians [71]. The hepatoprotective effects of OO, FO, and their blend on the hepatocytes of rats’ liver resulted in a substantial reasonable increase in rat weight as compared to the control groups. This finding supports its curative effects against negative weight loss associated with various diseases [68].

## 9. Conclusions

There is no single VO with composition levels suitable to meet the recommended levels of SFA, MUFA, PUFA, and the Ω-3 to Ω-6 PUFA ratio. Some oils are rich in SFAs (CO), some are flourished with Ω-3 PUFAs (FO), while others are dense in MUFA (OO), and most are majorly composed of Ω-6 PUFA (SFO). Besides being protective against various disease biomarkers, these differentiated FA types and selective qualities of VOs make them inappropriate when consumed alone in 100% of fat recommendations on a daily basis. Therefore, blending such VOs with differentiated FA types and selective qualities can serve as a cost-effective solution towards the health improvement of the whole population in general. This paper summarizes the latest available data on BOs from CO, FO, OO, and SFO, based on their contrasting FA profiles. The results from various studies go in favor of the blending of different contrasting VO in order to improve their nutritional, physiochemical, and therapeutical potential. This could ultimately help the oil industry to develop its production while serving a cost-effective role in the prevention of chronic CDs and NCDs. Future recommendations are advised on other oil varieties and/or other variable characteristics of VOs.

## Figures and Tables

**Table 1:** Nutritional and physiochemical profile of selected oils (coconut oil, flaxseed oil, olive oil, sunflower oil) (a: [21]; b: [30]; c: [31]; d: [32]; e: [24]; f: [20]; g: [33]; h: [34]; i: [35]; j: [36]; k: [37]; l: [38]; m: [39]; n: [40]; o: [41]).

Oil	FA Profile						Bioactive Components	Oxidative Stability			
	SFA		MUFA		PUFA			Acid Value	Peroxide Value	TBA Value	FFA
	FA	%	FA	%	FA	%					
**Coconut oil**	C8:0 (Caprylic)	5.85 ^a^, 10.4 ^b^, 2.76 ^c^, 7.39 ^k^	C16:1 (Palmitoleic)	-	C18:2n6c (Linoleic)	2.54 ^a^, 2.9 ^b^, 1.90 ^c^, 1.16 ^k^	Phenolic compounds = 132.15 mg/g; DPPH IC50 (µg/mL) ≥ 1000	0.40 ^a^, 0.24 ^i^, 0.48 ^j^, 0.24 ^j^ 15.71 mg KOH/g ^o^	0.61 ^a^, 8.8 ^j^	-	-
	C10:0 (Capric)	4.86 ^a^, 5.7 ^b^, 5.18 ^c^, 6.02 ^k^	C18:1n9c (Oleic)	8.11 ^a^, 6.5 ^b^, 7.24 ^c^, 5.54 ^k^							
	C12:0 (Lauric)	47.97 ^a^. 46.^b^, 49.57 ^c^, 49.61 ^k^									
	C14:0 (Myristic)	19.35 ^a^, 18.9 ^b^,21.12 ^c^, 18.44 ^k^	C20:1n9 (Eicosenoic)	-	C18:3n3c (Linolenic)	-					
	C16:0 (Palmitic)	8.80 ^a^, 8.6 ^b^, 9.26 ^c^, 8.44 ^k^									
	C18:0 (Stearic)	2.97 ^a^, 2.9 ^bk^	C22:1n9 (Eruic)	-							
	C20:0 (Arachidic)	-									
	C22:0 (Behenic)	-	C24:1	-	C20:2	-					
	C24:0 (Lignoceric)	-									
	TSFA	90.84 ^a^, 93.30 ^k^	TMUFA	7.24 ^a^, 5.54 ^k^	TPUFA	1.90 ^a^, 1.16 ^k^					
	SFA: MUFA: PUFA	35.1:3.1:1 ^a^									
	Ω-6/Ω-3	-									
**Flaxseed/Linseed oil**	C8:0 (Caprylic)	-	C16:1 (Palmitoleic)	0.03 ^a^,	C18:2n6c (Linoleic)	13.36 ^a^, 14 ^b^, 14.31 ^d^, 12.90 ^f^, 16.60 ^k^	α-Tocopherol = 29 mg/100 g moil ^g^, βTocopherol = 1.0 mg/100 gmoil ^g^, γ-Tocopherols = 1.0 mg/100 gmoil ^g^, Vitamin E = 7.50 ^l^, Phytosterol = 116.98	1.07 ^a^, 0.80 ^j^, 0.40 ^j^	1.40 ^a^, 8.5 meq/kg ^j^	-	-
	C10:0 (Capric)	-	C18:1n9c (Oleic)	19.29 ^a^, 25 ^b^, 17.30 ^d^, 20.3 ^f^, 18.20 ^k^							
	C12:0 (Lauric)	2.13 ^a^									
	C14:0 (Myristic)	0.99 ^a^, 0.03 ^b^	C20:1n9 (Eicosenoic)	0.12 ^k^							
	C16:0 (Palmitic)	5.79 ^a^, 6.5 ^b^, 6.7 ^f^, 4.54 ^k^									
	C18:0 (Stearic)	4.69 ^a^, 6.2 ^b^, 2.5 ^f^, 3.32 ^k^	C22:1n9 (Eruic)	-	C18:3n3c (Linolenic)	51.95 ^a^, 46.5 ^b^, 57.26 ^d^, 57.1 ^f^, 56.66 ^k^					
	C20:0 (Arachidic)	0.21 ^a^, 0.2 ^f^, 0.12 ^k^									
	C22:0 (Behenic)	1.56 ^a^, 0.11 ^k^	C24:1	-	C20:2	-					
	C24:0 (Lignoceric)	-									
	TSFA	15.37 ^a^, 10.24 ^d^, 9.43 ^f^, 8.23 ^k^	TMUFA	19.29 ^a^, 20.3 ^f^, 18.32 ^k^	TPUFA	65.31 ^a^, 70 ^f^, 73.45 ^k^					
	SFA: MUFA: PUFA	1:1.2:4.3 ^a^									
	Ω-6/Ω-3	0.30 ^k^									
**Olive oil**	C8:0 (Caprylic)		C16:1 (Palmitoleic)	1.11 ^a^, 0.1 ^b^, 3 ^f^, 0.12 ^g^, 0.87 ^k^	C18:2n6c (Linoleic)	9.59 ^a^, 4.8 ^b^, 7.12 ^d^, 11.4 ^f^, 15.26 ^g^, 7.79 ^k^	Vitamin E = 4.40 mg ^l^, Phytosterol = 54.02 g ^l^ 3,4-dihydroxy benzoic acid = 9.8 ppm ^m^, 0.52 mg/kg ^n^ Tyrosol = 2.8 ppm ^m^, 0.8 mg/kg ^n^ 4-hydroxy benzoic acid = 3.9 ^m^ m-coumaric acid = 3 ppm ^m^ Oleuropein = 5.4 ppm ^m^, 21.9 mg/kg ^n^ Luteolin = 222 ppm ^m^, 2.5 mg/kg ^n^ A tocopherol = 324.6 ppm ^m^ Total phenolic compounds = 166.7 mg/kg ^n^	0.49 ^a^	0.81 ^a,^ 17.9 ^m^	-	0.42 ^g^, 0.3% ^m^
	C10:0 (Capric)	-	C18:1n9c (Oleic)	71.15 ^a^, 75.7 ^b^, 72.06 ^c^, 69.1 ^f^, 56.74 ^g^, 75.31 ^k^							
	C12:0 (Lauric)	-									
	C14:0 (Myristic)	0.10 ^g^	C20:1n9 (Eicosenoic)	0.21 ^gk^							
	C16:0 (Palmitic)	14.06 ^a^, 11 ^b^, 13.2 ^f^, 17.74 ^g^, 11.55 ^k^									
	C18:0 (Stearic)	3.02 ^a^, 4.3 ^b^, 4 ^f^, 2.71 ^g^, 2.98 ^k^	C22:1n9 (Eruic)	-	C18:3n3c (Linolenic)	0.99 ^a^, 0.3 ^b^, 0.59 ^d^, 1.2 ^f^, 0.77 ^g^, 0.72 ^k^					
	C20:0 (Arachidic)	0.6 ^f^, 0.44 ^g^, 0.37 ^k^									
	C22:0 (Behenic)	0.11 ^g^, 0.10 ^k^	C24:1	-	C20:2	-					
	C24:0 (Lignoceric)	0.5 ^f^, 0.12 ^g^									
	TSFA	17.08 ^a^, 15.53 ^d^, 18.9 ^f^, 21.33 ^g^, 15.05 ^k^	TMUFA	72.26 ^a^, 72.03 ^f^, 62.64 ^g^, 76.44 ^k^	TPUFA	10.58 ^a^, 12.6 ^f^, 16.03 ^g^, 8.51 ^k^					
	SFA: MUFA: PUFA	1.6:6.8:1 ^a^									
	Ω-6/Ω-3	19.81 ^g^, 10.95 ^k^									
**Sunflower oil**	C8:0 (Caprylic)	-	C16:1 (Palmitoleic)	0.09 ^e^, 0.14 ^g^,	C18:2n6c (Linoleic)	62.67 ^a^, 64.1 ^b^, 54.17 ^c^, 62.58 ^d^, 64.95 ^e^, 65.76 ^g^, 62.97 ^k^	α-Tocopherol = 47 mg/100 gmoil ^g^, βTocopherol = 0.3 mg/100 gmoil ^g^, γ-Tocopherols = 0.7 mg/100 gmoil ^g^, Vitamin E = 10.9 ^l^, Phytosterol = 74.45 g ^l^	1.06 ^a^, 0 ^h^, 0.51 ^i^, 0.48 ^j^, 0.24 ^j^	1.02 ^a^, 0.0823 ^h^, 8 meq/kg ^j^	0.0573 ^h^	1.70 ^g^
	C10:0 (Capric)	-	C18:1n9c (Oleic)	26.21 ^ab^, 24.77 ^c^, 24.61 ^d^, 23.41 ^e^, 19.93 ^g^, 26.28 ^k^	C18:3n3c (Linolenic)	0.29 ^a^, 0.79 ^b^, 5.16 ^c^, 0.09 ^d^, 0.10 ^e^, 0.11 ^g^, 0.38 ^k^					
	C12:0 (Lauric)	-									
	C14:0 (Myristic)	0.12 ^g^, 0.07 ^e^	C20:1n9 (Eicosenoic)	0.10 ^e^, 0.36 ^g^, 0.15 ^k^	C20:2	-					
	C16:0 (Palmitic)	6.97 ^a^, 5.5 ^b^, 6.43 ^c^, 6.16 ^e^, 9.43 ^g^, 5.83 ^k^	C22:1n9 (Eruic)	-							
	C18:0 (Stearic)	3.28 ^a^, 4.2 ^b^, 3.69 ^c^, 4.29 ^e^, 4.68 ^g^, 3.24 ^k^	C24:1	-							
	C20:0 (Arachidic)	0.57 ^a^, 0.32 ^c^, 0.27 ^e^, 0.28 ^g^, 0.21 ^k^									
	C22:0 (Behenic)	0.51 ^e^, 0.79 ^g^, 0.59 ^k^									
	C24:0 (Lignoceric)	0.21 ^g^, 0.19 ^k^									
	TSFA	10.82 ^a^, 15.90 ^b^, 11.34 ^d^, 12.93 ^g^, 10.16 ^k^	TMUFA	26.21 ^a^, 24.77 ^b^, 21.20 ^g^, 26.50 ^k^	TPUFA	62.96 ^a^, 59.33 ^b^, 65.87 ^g^, 63.34 ^k^					
	SFA: MUFA: PUFA	1.6:6.8:1 ^a^									
	Ω-6/Ω-3	939 ^g^, 594.71 ^k^									

**Table 2:** Nutritional and physiochemical profile of various blends of selected oils (coconut oil, flaxseed oil, olive oil, sunflower oil).

Oil Source	Blending Type	Blending Ratios	Methodology	Results	References
SFO, FO	Binary	10:90, 20:80, 30:70, 40:60, 50:50, 60:40, 70:30, 80:20, 90:10	FA composition, tocopherols, carotenoids, functional composition	FO blending in SFO resulted in increased ALA and carotenoids, and balanced resultant oil composition	[57]
POL-DAG, VCO	Binary	10:90, 20:80, 30:70, 40:60, 50:50, 60:40, 70:30, 80:20, 90:10	FA, AG composition, functional groups, TS, solid fat content, IV	Blending of POL-DAG with VCO enhanced all of the techno-functional properties of the oil	[58]
RBO, PAO, FO	Ternary	50:40:10, 55:40:5, 60:30:10, 65:30:5, 70:20:10, 75:20:5	FA composition, tocopherols, peroxide value, Acid value	Enhanced OS	[59]
SFO, PSO	Binary	90:10, 85:15, 80:20	Total phenolics, total carotenoids, tocopherols, FA composition, and storage	Increased OS in BO as compared to only SFO	[23]
LO, SBO, MO, CO, OO, SFO	Binary	20:80, 30:70, 40:60, 50:50, 60:40, 70:30 and 80:20 (*v*/*v*)	FA composition, Chemical properties, and OS of blends	FA ratios (SFA:MUFA:PUFA) of 1.5:1:3.1 for LO and CO (80:20), 1:1.4:4.6 for LO and SBO (20:80), and 1:1.9:3.4 for LO and OO (80:20) were found healthier. Poor storage quality observed due to high PUFA in (LO:SBO, LO:SFO & LO:MO)	[21]
FO and POL	Binary	Three different percentages of FO (20, 10 and 5 *v*/*v*) blended with the POL.	FA composition, Chemical properties, the 9-month storage stability of blends	Blending improved Ω-6:Ω-3 ratio and OS and TS on nine-month storage	[48]
CAO, OO, SSO	Binary	CAO–OO and CAO–SSO (90:10, 70:30, and 50:50)	Rheology, viscosity, Shear-thinning fluids, before and after cooking	Blending did not affect the shear thinning and viscosity of oils	[60]
VCO, POL	Binary	10:90, 20:80, 30:70, 40:60, 50:50	Rheological attributes and storage period	Resultant blends were trans-free, sheer thinning and had high melting points	[27]
CO, PNO, PAO, GNO	Binary	50:50 % mixed for 1 h in a blender to form uniform blends	Color, FFA, SA, PV, SV, IV & product development	CO, along with its blends, was found best, yielding favorable effects with minimal increases in PV, FFA, IV, and SV	[26]
SFO, LO, MO, PKO, WGO, MTO	Ternary	Blend A: MO, PKO, MTO; Blend B: SFO, LO, MTO; Blend C: SFO, WGO, MTO	Modeling the blend composition by “brute force” method as the objective function (Ω-6 to Ω-3 as 5:1)	The blending of edible oil is justified for formulating the oils with desired FA composition and Ω-6 to Ω-3 ratios	[61]
PAO, CO, RBO	Not Reported Yet	Mixture 1 and mixture 2 of PAO, CO, RBO	Effect of deep frying for 12 min at 150 °C and 170 °C in two cycles	The blending of VOs results in lower acrylamide content in deep-fried food	[62]
SFO, CO	Binary	50:50, 70:30 (%)	FA composition, AV, FFA, OSI	Blends revealed increased OS and antioxidant potential by DPPH tests due to raised phenolic compounds	[35]
OO, SFO, CRO	Ternary/Ω-6/Ω-3 ratios	Oil mixtures with 2, 3, 4, and 5 Ω-6/Ω-3 ratios	OS and thermal stability, FA composition, tocopherol, physicochemical properties	Blends resulted in high OS, high antioxidant content, optimal Ω-6/Ω-3 ratios, with good functional characteristics and health benefits	[33]
SFO, LO, GSO, CO	Binary	Blends of SFO with LO, GSO, and CO with ratios (*v*/*v*): 90:10; 80:20; 70:30	The lovage leaves were added in oils and oil blends by extraction of chlorophyll and other phytochemicals directly in oils to increase stability	The extracted phytochemicals reduced the AV and PV. The findings support oil blending, particularly for reducing acidity, as well as fresh herbal addition for reducing autoxidation processes, both of which improve the quality of edible VOs	[36]
LO, CTO, CO	Binary & Ternary	LO:CTO 1:1; LO:CO 1:1; LO:CTO:CO 2:1:1	Accelerated storage test at 60 °C for 20 days; FA composition; phenolic compounds; antioxidant activity	Decreased formation of degradation compounds (especially in LA), predomination of PUFA and ALA, less reduction of phytosterols and tocopherols during storage (especially LC with 95.1% of phytosterols, and LA, with 90.81% of tocopherols). Blending with CTO and CO increased LO stability, which, in turn, raised the levels of CO bioactive compounds	[63]
OO, SSO, LO	Ternary	Three ratios of OO:SSO:LO, 65:30:5; 60:30:10 55:30:15	Chemical, nutritional, rheological properties, AV, PV, rancimat test, FA composition	Blending could balance Ω-6:Ω-3 ratio; OS and nutritional properties. Rheological data showed that these oil blends followed Newtonian behavior at 4 °C and 25 °C	[20]
SFO, SBO, FO, MO, EVOO & others	Binary	B1 (55 SFO; 45 WO); B2 (75 SFO; 25 FO); B3 (60 SFO:40 Camelina)	FA composition, OS at 20 ± 2 °C with free exposure to light and air, Ω-6/Ω-3 optimal ratio	Healthy Ω-3:Ω-6 ratios of B1 = 1:10; B2 = 1:3.5; B3 = 1:3.3 were obtained. SFO and FO blend was found with the least OS	[32]
BCO, SFO	Binary	Blends (5%, 10% and 20%, *w*/*w*) of cold-pressed BCO with SFO	-	Increased TS at high temperatures; raised α-tocopherol and thymoquinone in blends; 80:20 SFO:BCO blend showed the highest OS among oil blends	[24]
OO, LO, SAO	Ternary/Ω-6/Ω-3 ratio	3 blends (A, B, C) of different proportions of oils, EFA and Ω-6/Ω-3 ratio	FA composition, OS, tocopherols, phytosterols, and sensory acceptance	Blend C (85% OO, 3% LO, 12% SAO) presented 44% higher EFA, Ω-6/Ω-3 ratio twice lowered, raised levels of sterols & tocopherols, good OS and sensorial acceptation	[64]
OO, SSO, FO	Ternary	Three ratios of OO:SSO:LO, 65:30:5; 60:30:10, 55:30:15	Chemical, nutritional, rheological properties, AV, PV, rancimat test, FA composition	The addition of FO revealed improved ratios of EFA, greatest phenolic concentration, which decreased during storage. The PV of all samples increased significantly after storage	[19]

**Table 3:** Therapeutic potential of blends of selected oils (coconut oil, flaxseed oil, olive oil, sunflower oil).

Oil Source	Nutraceutical Prominence	Methodology	Results	References
FO, POL	THP-1 cell line, FA uptake and inflammatory markers	THP-1 (human monocytic leukemia cell line), cultured in RPMI 1640 medium containing 10% FBS, incubated at 37 °C in a humidified incubator containing 5% CO2, seeded in 24 W ELISA plate.	FA profiles of the cells treated with these blends showed uptake of Ω-3 FA ALA. These blends lowered inflammatory TNF-α level without affecting cell survival.	[48]
CO, CNO blends with linoleic acid (LA) levels of very low, low, moderate, High	Obesity, body fat deposition and other metabolic biomarkers	8-week efficacy trial on rats to investigate body fat deposition and selected metabolic biomarkers	The extremely low-LA, high- MCFA diet resulted in less body weight increase than the high-LA diet. The high-LA diet significantly increased body fat deposition compared to the very low-LA and low-LA diets.	[65]
RBO, FO, SSO	Lipid and cardiac biomarkers	Randomised controlled study on 143 borderline hypercholesterolemic volunteers aged 50 to 70 with BMI ≤ 27.5.	Significant reductions in TC, LDL, TAG, apoB, TC to HDL and apoB to apoA1 ratios, systolic & diastolic BP, and serum glucose, as well as a small significant increase in body weight.	[66]
Mixed plant oils (MPO): 10% CO, 15% CNO, 15% LO, 15% PNO, 20% PAO, and 25% SBO	Performance, antioxidant capacity, serum immunity, and intestinal morphology	28 days efficacy trial on 108 piglets with soybean oil as control and MPO as a study group.	MPO increased average daily weight gain and feed efficiency, improved serum peroxide dismutase (SOD), higher serum IgG, glutathione peroxidase contents, villus height in duodenum and jejunum, and apparent total tract digestibility of ether extract.	[67]
(VCO and OO with Vitamin E	DM and oxidative damage to beta cells after alloxan injection.	Hyperglycemic Rattus norvegicus administered mixture of VCO + Vit E, and OO + Vit E for 4 weeks, and its effects on the liver. 0.1 mL/BW of each VCO + Vit E and OO + Vit E was injected.	VCO and OO are both capable of lowering blood glucose levels without altering the shape of hepatocyte cells or the hepatosomatic index.	[45]
SFO, CO	50:50, 70:30 (%) Blends for antioxidant activity test	Free radical DPPH (2,2-diphenyl-1-picrylhydrazyl) was performed on samples at concentrations of 10–1000 µg/mL.	The combination of CO and SFO boosted antioxidant capacity, as shown by DPPH tests.	[35]
OO, SFO, CAO, CNO, PNO, LRO	Effect of high oleic acid (50.93%) and ALA (5.41%) on HTN, CVDs, and body weight	12 weeks trial on 90 male Wistar rats divided into 3 main groups (blend, LRO, PNO) and 3 subgroups (low, middle and high fat)	Blend oil with a low Ω-6/Ω-3 PUFA ratio of 6:1 prevented and controlled cardiovascular disease, weight gain, body fat deposition and inflammatory biomarkers.	[4]
OO, CNO, FO	Hepatoprotictive effects of OO, FO and their blend	One month study on 60 rats, injected with CCl4 in paraffin oil for 2 weeks to develop chronic hepatitis in the liver.	Reduction of total cholesterol, improvements in liver, significant amelioration in CCl4 induced necrosis and infiltration of lymphocytes.	[68]
CO, OO, SFO, LO	Growth and feed efficacy	9–12 week trial on Nile tilapia growth at two temperatures: optimal (28 °C) or suboptimal (22 °C)	Plant oil mixes had same effect on growth and feed efficiency of Nile tilapia at either 28 °C or 22 °C rearing temperatures	[69]
SBO, LO (1:1)	Serum lipid metabolic responses associated with metabolic disorders in mammals	70-day trial on 270 large yellow croaker fish (Larimichthys crocea)	VO blend strongly affected serum lipid profiles, especially ratio of triglyceride:phosphatidylcholine (TAG:PC)	[70]
LO with curcumin	Increased formation of DHA from ALA by activating the enzymes FADS2 and elongase 2	60 days efficacy study on rats.	Curcumin and LO in phospholipid-based nanoemulsions dramatically increased DHA levels in serum, liver, heart, and brain lipids in rats.	[71]

## Data Availability

Not applicable.

## Abbreviations

**Oils:** AG (Acylglycerol); BCO (Black cumin oil); BO (Blended Oil); CO (Coconut Oil); CAO (Canola Oil); CNO (Corn Oil); CRO (Cress Oil); CTO (Cotton Oil); DHA (Docosahexaenoic Acid); EVCO (Extra Virgin Coconut Oil); EVOO (Extra Virgin Olive Oil); FO/LO (Flaxseed Oil/Linseed Oil); GNO (Groundnut Oil); GSO (Grapeseed Oil); (LRO) Lard Oil; MO (Mustard oil); MTO (Milk thistle Oil); OO (Olive Oil); PAO (Palm Oil); PKO (Pumpkin Oil); POL (Palm Olein); POL-DAG (Palm Olein-based Diacylglycerol); PSO (Pomegranate Seed Oil); PNO (Peanut Oil); RBO (Rice Bran Oil); SAO (Safflower Oil); SBO (Soyabean Oil); SSO (Sesame Oil); SFO (Sunflower Oil); VO (Vegetable Oil); VCO (Virgin Coconut Oil); WGO (Wheat germ oil).

**Others:** ALA (Alpha Linolenic Acid); AV (Acidic Value); CCL4 (Carbon tetrachloride); FFA (Free Fatty Acids); MCFA (Medium-chain Fatty Acid); OA (Oleic Acid); OS (Oxidative Stability); TS (Thermal Stability); SA (Spread Ability), PV (Peroxide Value), SV (Saponification Value), IV (Iodine Value); OSI (Oil Stability Index); HTN (Hypertension); CVD (Cardiovascular Diseases); DM (Diabetes Mellitis); TC (Total Cholesterol); LDL (Lower Density Lipoprotein); TAG (Triacylglycerols); HDL (High-density Lipoprotein) and Apo (Apolipoprotein).
